# Physiologic Measures of Animal Stress during Transitional States of Consciousness

**DOI:** 10.3390/ani5030380

**Published:** 2015-08-07

**Authors:** Robert E. Meyer

**Affiliations:** College of Veterinary Medicine, Mississippi State University, Mississippi State, MS 39762, USA; E-Mail: robert.meyer@msstate.edu; Tel.: +1-662-325-1453; Fax: +1-662-325-4011

**Keywords:** consciousness, anesthesia, euthanasia, stress markers, heart rate, ECG, EEG, BOLD MRI, c-fos

## Abstract

**Simple Summary:**

The humaneness, and therefore suitability, of any particular agent or method used to produce unconsciousness in animals, whether for anesthesia, euthanasia, humane slaughter, or depopulation, depends on the experience of pain or distress prior to loss of consciousness. Commonly reported physiologic measures of animal stress, including physical movement and vocalization, heart rate and ECG, electroencephalographic activity, and plasma and neuronal stress markers are discussed within this context.

**Abstract:**

Determination of the humaneness of methods used to produce unconsciousness in animals, whether for anesthesia, euthanasia, humane slaughter, or depopulation, relies on our ability to assess stress, pain, and consciousness within the contexts of method and application. Determining the subjective experience of animals during transitional states of consciousness, however, can be quite difficult; further, loss of consciousness with different agents or methods may occur at substantially different rates. Stress and distress may manifest behaviorally (e.g., overt escape behaviors, approach-avoidance preferences [aversion]) or physiologically (e.g., movement, vocalization, changes in electroencephalographic activity, heart rate, sympathetic nervous system [SNS] activity, hypothalamic-pituitary axis [HPA] activity), such that a one-size-fits-all approach cannot be easily applied to evaluate methods or determine specific species applications. The purpose of this review is to discuss methods of evaluating stress in animals using physiologic methods, with emphasis on the transition between the conscious and unconscious states.

## 1. Introduction

Documenting procedural humaneness in animals during transitional states of consciousness can be difficult because as humans we can never fully know or understand the subjective experiences of animals. This problem has vexed veterinary medicine since 1847 when, after administering the then newly-described diethyl ether anesthetic to dogs and cats, the eminent British veterinarian Edward Mayhew concluded:

“The results of these trials are not calculated to inspire any very sanguine hopes. We cannot tell whether the cries emitted are evidence of pain or not; but they are suggestive of agony to the listener, and, without testimony to the contrary, must be regarded as evidence of suffering. The process, therefore, is not calculated to attain the object for which in veterinary practice it would be most generally employed, namely, to relieve the owner from the impression that his animal was subjected to torture. In another light, it is not likely to be of much practical utility.” [1].

Fortunately, the further development of veterinary anesthesia continued despite Dr. Mayhew’s initial misgivings. 

Unconsciousness, whether induced by anesthesia or methods associated with euthanasia, humane slaughter, and depopulation methods, is the singular result of three potential basic mechanisms: (1) direct pharmaceutical effect on the central nervous system; (2) hypoxia; and (3) physical disruption of brain activity; death subsequently follows as the circulatory and respiratory centers fail, or as hypoxia or reduced pH render intracellular processes nonfunctional. However, as loss of consciousness occurs at substantially different rates with various methods, the suitability of any particular agent or method depends largely on whether pain or distress is experienced prior to loss of consciousness. 

Stress and the resulting responses have been divided into three phases [2]. Eustress results when harmless stimuli initiate adaptive responses that are beneficial to the animal. Neutral stress results when the animal’s response to stimuli causes neither harmful nor beneficial effects to the animal. Distress results when an animal’s response to stimuli interferes with its well-being and comfort [3]. Distress may be created by conditions experienced prior to loss of consciousness (e.g., transport conditions, environment, or restraint), or by conditions under which methods are applied (e.g., gradual displacement application of gas/vapor or immersion into a high gas/vapor concentration [4]). 

It is important to note stress responses to agents or conditions may be highly variable between phyla (e.g., avian *vs*. mammalian species), between species within the same phyla, and even within species. Simply placing Sprague-Dawley rats into an unfamiliar exposure chamber containing room air produces arousal, if not distress [5]. Pigs are social animals and prefer not to be isolated from one another; consequently, moving them in groups, rather than lining them up single-file, improves voluntary forward movement, reduces handling stress, and reduces electric prod use [6]. Gradual-fill inhaled anesthesia or euthanasia methods may be less stressful to rats than more rapid displacements or immersion [7]. Distress may manifest behaviorally (e.g., overt escape behaviors, approach-avoidance preferences [aversion]) or physiologically (e.g., movement, vocalization, changes in electroencephalographic activity, heart rate, sympathetic nervous system [SNS] activity, hypothalamic-pituitary axis [HPA] activity), such that a one-size-fits-all approach cannot be easily applied to evaluate humaneness of methods or determine specific species applications. 

While methods to assess pain and distress have come a long way since 1847, we still lack simple, effective, and incontrovertible means to objectively evaluate the animal experience during the transition to unconsciousness. The purpose of this review is to discuss methods of evaluating stress in animals using physiologic methods, with emphasis on the transition between the conscious and unconscious states.

## 2. Literature Review

### 2.1. Movement, Vocalization, and Reflex Activity

Pain is defined as a conscious perception. Pain is quite subjective in the sense that individuals can differ in their perceptions of intensity as well as in their physical and behavioral responses. The International Association for the Study of Pain (IASP) describes pain as:

“An unpleasant sensory and emotional experience associated with actual or potential tissue damage, or described in terms of such damage. Activity induced in the nociceptor and nociceptive pathways by a noxious stimulus is not pain, which is always a psychological state, even though we may well appreciate that pain most often has a proximate physical cause.” [8].

Based on mammalian models, the perception of pain requires nerve impulses from peripheral nociceptors to reach an awake, functioning cerebral cortex and associated subcortical brain structures. Impulses from peripheral nociceptors are conducted by primary afferent fibers to either the spinal cord or the brainstem and two general sets of neural networks. Reflex withdrawal and flexion in response to nociceptive input is mediated at the spinal level, while ascending nociceptive pathways carry impulses to the reticular formation, hypothalamus, thalamus, and cerebral cortex (somatosensory cortex and limbic system) for conscious sensory processing and spatial localization. This distinction is important, in that movements observed in response to nociception can be due to spinally-mediated reflex activity (unconscious), cerebral cortical and subcortical processing (conscious), or a combination of the two. 

In humans, onset of anesthetic-induced unconsciousness has been functionally defined by loss of appropriate response to verbal command; in animals, loss of consciousness is functionally defined by loss of the righting reflex (LORR), also called loss of position (LOP) [9,10,11]. This definition, introduced with the discovery of general anesthesia over 160 years ago, is still useful because it is an easily observable, integrated whole-animal response which is applicable to a wide variety of species.

Although the perception of pain requires a conscious experience, defining consciousness, and therefore the ability to perceive pain, across species is difficult. Amnesia, defined as loss of memory function in which old memories cannot be remembered or new memories cannot be formed, is a defining characteristic of anesthesia, in that with sufficient quantities, all anesthetics are capable of producing a state of amnesia where new memories cannot be formed [12]. Unconsciousness, defined as loss of individual awareness, occurs when the brain’s ability to integrate information is blocked or disrupted. Anesthetics produce unconsciousness either by preventing integration (blocking interactions among specialized brain regions) or by reducing information (shrinking the number of activity patterns available to cortical networks) received by the cerebral cortex or equivalent structure or structures; Further, the abrupt loss of consciousness that occurs at a critical concentration of anesthetic implies nonlinear collapse of the integrated workings of the interconnected neural states underlying consciousness [13,14]. 

Physical disruption of brain activity, whether by gunshot, captive bolt, cerebral electrocution, blunt force trauma, or maceration, produces instantaneous unconsciousness by destroying or rendering nonfunctional brain regions responsible for cortical integration; death quickly follows when the midbrain centers controlling respiration and cardiac activity fail. Signs of effective stun resulting in unconsciousness in cattle include immediate collapse (LORR/LOP) and a several-second period of tetanic spasm, followed by slow hind limb movements of increasing frequency [15,16,17]; however, there is species variability in this response. Signs of effective electrocution are loss of righting reflex, loss of eye blink and moving object tracking, extension of the limbs, opisthotonos, downward rotation of the eyeballs, and tonic (rigid) spasm changing to clonic (paddling) spasm, with eventual muscle flaccidity [16,18]. The corneal reflex will be absent with effective mechanical and electrical stunning [19]. Generalized seizures, while unpleasant to watch, are associated with complete loss of awareness and consciousness [20]. 

While purposeful or directed escape behaviors should not be observed during the transition to unconsciousness with anesthesia or humane killing methods, vocalization or physical movement is often interpreted as unequivocal evidence of consciousness. Physical responses observed during induction and maintenance of anesthesia, including reflex activity, eye globe rotation, general muscle tone, and autonomic activity have been historically taught and utilized in both humans and veterinary patients as ‘signs of consciousness’ and to monitor depth of anesthesia. However, these physical responses are now considered to be unreliable when assessing depth of anesthesia as interpretation is based on subjective evaluation of autonomic reflexes and muscle activity; further, these traditional physical responses are affected in substantially different ways by different anesthetics [21]. Similarly, any movement during anesthesia and surgical procedures has traditionally been interpreted as indicating an animal is ‘underanesthetized’. Emerging evidence now suggests that, in unconscious anesthetized animals, movement in response to noxious stimulation is due to spinally mediated reflex activity and abolished primarily by means of anesthetic action in the spinal cord, rather than the cerebral cortex [10]. Human and animal studies confirm that amnesia is produced, and conscious awareness is blocked, at less than half the anesthetic concentration required to abolish physical movement [10]. Physical movements can even occur in the presence of electroencephalograhic burst suppression, a drug-induced or brain damaged pattern always associated with unconsciousness; indeed, spontaneous and often complex movement can occur in unconscious, decerebrate, or spinally transected humans and animals due to neural circuits at the level of the brainstem and spinal cord not requiring connection with the cerebral cortex [10]. Once consciousness is lost, subsequent activities, such as seizures, vocalization, reflex struggling, breath holding, and tachypnea, can be attributed to the “excitement” phase or anesthesia stage 2, which by definition lasts from loss of consciousness to the onset of a regular breathing pattern [22,23]. Thus, vocalization and non-purposeful movements observed following LORR/LOP are not necessarily signs of conscious perception by an animal.

Accelerometers have been used to document animal movement when the animal cannot be directly observed, such as during studies of water-based foams for poultry depopulation [24,25]. Although the onset of neuromuscular spasms does not necessarily indicate unconsciousness or insensibility, the complete cessation of neuromuscular spasms appears to be associated with brain death [26]. 

### 2.2. Heart Rate and Electrocardiography

Although heart rate has traditionally been utilized as a clinical sign in both humans and veterinary patients to monitor depth of anesthesia, as noted in Section 2.1, it is presently considered to be unreliable for this purpose as a wide range of conditions or situations can lead to similar changes [21]. Heart rate is controlled by the autonomic nervous system; increased sympathetic nervous system (SNS) activity results in an increased heart rate (tachycardia), while decreased heart rate (bradycardia) can be due to SNS depression or an increase in parasympathetic NS activity. Tachycardia and bradycardia are nonspecific in that several possible causes can result in the same net change in heart rate. Unconscious animals may not feel pain, but they will show autonomic NS responses to nociception such as toe pinch or skin incision. A differential list for tachycardia in the anesthetized patient could include:

- Sympathetic stimulation (e.g., “too light” or pain if awake)
- Hypotension
- Hypovolemia
- Hypoxemia (early) or anemia
- Hypercarbia
- Hyperthermia
- Drugs (e.g., ketamine, propofol, thiobarbiturates, antimuscarinics)

Similarly, a differential list for bradycardia could include:

- Excessive sympathetic depression (e.g., “too deep”)
- Increased vagal tone (due to laryngeal sensory stimulation, surgical activity, or autonomic imbalance)
- Hypothermia
- Hyperkalemia
- Elevated intracranial pressure
- Hypoxemia
- Hypercarbia
- Drugs (e.g., opioids, alpha-2 agonists, acetylcholine)

Electrocardiography represents a 2 dimensional recording at the body surface of electrical fields generated by the heart. As such, the electrocardiogram (ECG) provides a continuous waveform representing the summation of cardiac electrical vectors within a given axis or plane established by the skin surface electrode placement [27]. The ECG X-axis determines the time component for each cardiac depolarization-repolarization cycle, while the Y-axis represents cardiac action potential vector amplitude in mV. With appropriate standardization of lead placement, body position, and input voltage, a diagnostic ECG can be used to determine cardiac chamber size by measuring the amplitude of Y-axis deflections in the standard and augmented lead placements. Heart rate is determined from the Y-axis ECG deflections, typically the R wave, during a period of time as measured on the X-axis. Cardiac conduction pathway disturbances can be also be determined through measurement of the ECG waveform components relative to the X-axis. Portable strap-on exercise monitors report heart rate based on cardiac electrical activity as determined from the R wave deflections. These monitors utilize conductive rubber skin electrodes retained under a thoracic belt rather than the standardized lead placements as utilized in the diagnostic ECG. Movement artifact can be problematic.

While heart rate and the ECG are often reported as conservative estimates of time of death, neither heart rate nor ECG provide information as to the state of consciousness. As noted in the section on plasma stress responses, responses to systemic stressors associated with immediate survival, such as hypoxia and hypercapnia, are likely relayed from brainstem nuclei and are not associated with higher order CNS processing and conscious experiences [28]. In addition, interpretation of heart rate and ECG obtained during inhaled or injectable methods is confounded by continued exposure during the period between loss of consciousness and death. The presence of cardiac electrical activity does not imply effective cardiac pumping as the ECG (or in the case of exercise monitors, heart rate derived from the ECG) tells us nothing about the mechanical status of the heart [27]; indeed, pulseless electrical activity (previously called electromechanical dissociation) is a commonly observed phenomenon observed during cardiopulmonary resuscitation following cardiac arrest [29]. Thus, animals with continuing electrical activity should be monitored at intervals until death can be confirmed. 

### 2.3. Electroencephalography, Blood Oxygen Level Dependent MRI, Positron Emission Tomography

Measurements of brain electrical function, such as electroencephalogram (EEG), bispectral EEG analysis (BIS), and visual and auditory evoked potentials (VEP; AEP), have been used in attempts to define and quantify the unconscious state in man and animals [21]. In humans, the issue is intraoperative awareness under anesthesia; in animals, the problem is procedural humaneness relative to onset of unconsciousness. The problem is even more complex in animals where we cannot question them directly and must infer from their actions and responses. While measures of brain electrical activity are often applied in this context, these methods are currently acknowledged to be limited in their ability to provide definitive answers as to onset of either human or animal unconsciousness.

As previously discussed, onset of anesthetic-induced unconsciousness in animals is functionally defined by LORR/LOP. The usefulness of LORR/LOP as an easily observable proxy for loss of animal consciousness was recently reinforced when a reduction in alpha: Delta brain wave ratios was found to coincide with LOP in chickens [30,31]. Similarly, nictitating membrane activity was found to be a conservative indicator of brain death as defined by isoelectric EEG spectral activity in layer hens and turkeys [32].

However, EEG is not a direct measure of consciousness—it measures brain electrical activity, and while that activity changes with levels of consciousness, EEG cannot provide definitive answers as to precise onset of human unconsciousness using the current state of the art. Although consciousness must vanish at some level between behavioral unresponsiveness and the induction of a flat EEG (indicating the cessation of the brain’s electrical activity and brain death), current EEG-based brain function monitors are acknowledged to be limited in their ability to directly indicate presence or absence of unconsciousness, especially around the transition point [13,33]; also, it is not always clear which EEG patterns are indicators of activation by stress or pain [34]. 

Methods of EEG signal analysis can profoundly change the conscious/unconscious state interpretation. In isoflurane-anesthetized rats, a slowing of frequency and an increase in delta power similar to that reported in other species and humans was observed at LORR; turning the rats onto their sides, however, produced EEG signals quite similar to the awake resting state, that is, the high amplitude slow wave activity changed to low amplitude fast activity that lasted several seconds. This occurred regardless of whether the rat was able to right itself or not; further, paw pinch and tail clamp responses produced similar EEG signs of activation even during deep anesthesia when burst suppression dominated the spontaneous EEG. Waveform EEG analysis using chaos analysis and chaotic attractor shape was far better at discerning between these awake-like signals, at loss and return of righting responses, than was fast Fourier transformation analysis [35]. 

The specific conditions under which the EEG is obtained may also influence interpretation. In humans administered propofol, dexmedetomidine, or sevoflurane, EEG reactivity, defined by the authors as transient changes in electrical brain activity following external stimulation, differed substantially at the same clinical endpoint (in this case, nonresponsiveness to verbal command) [36]. This led the authors to conclude that it may be difficult to utilize EEG reactivity for depth of anesthesia monitoring [36]. This finding may have profound implications for the use of EEG for evaluation of animal consciousness in that EEG observations made under anesthesia may not be directly comparable between different anesthetics or physical methods. 

A recent editorial in a human anesthesia journal neatly sums up the issues:

“The electroencephalogram has been the Holy Grail of anesthetic depth monitoring for more than half a century but has fallen on hard times lately, largely because the focus of the dialog changed from electroencephalogram as a monitor of “depth” to one of intraoperative awareness… consciousness and intraoperative awareness are neurobiologically exceedingly complex phenomena. This makes these states difficult to capture or evaluate with electroencephalography, no matter the parameter or sophistication of the processing algorithm. Recent studies examining the efficacy of the electroencephalogram bispectral index for minimizing the risk of intraoperative awareness confirm as much.” [37].

Evoked potentials measure the electrophysiologic responses of the nervous system to a variety of stimuli. The visual evoked potential (VEP) tests the function of the visual pathway from the retina to the occipital cortex. It measures the conduction of the visual pathways from the optic nerve, optic chiasm, and optic radiations to the occipital cortex. Although loss of VEPs is associated with brain death, visual cortex neurons remain responsive to flash stimulation under desflurane anesthesia [38], and reduction in flash-induced gamma oscillations in rat visual cortex is not a unitary correlate of anesthetic-induced unconsciousness [39]. The auditory evoked potential (AEP) measures the functioning of the auditory nerve and auditory pathways in the brainstem. Large inter-individual variations in AEP and BIS analysis make it impossible to discriminate subtle changes of clinical state of consciousness in real time during propofol anesthesia [40].

Similarly, blood oxygen level-dependent MRI imaging (BOLD) is a multifactorial surrogate for cerebral blood flow or cerebral blood volume, both of which are actual measures of neural activity. BOLD MRI imaging exploits the presence of deoxyhemoglobin in tissues as a contrast media and provides *in vivo* maps of changes in brain activity triggered by pharmacological or physiological events. While a BOLD MRI response is generally a good surrogate measure of neuronal activity, it is based on hemodynamic changes which may not reflect actual neural activity patterns, especially during anesthesia or under pharmacological challenge; rather, observed effects may be directly due to drug effects, or indirectly through changes in autonomic activity, blood pressure, cardiac output, or respiration [41,42]. In contrast, imaging of cerebral blood flow using positron emission tomography has been used in human volunteers during manipulation of consciousness with anesthetics combined with response to verbal command [14]; application of this method to explore the transition between the conscious and unconsciousness state in animals will be difficult.

Decapitation and cervical dislocation as physical methods of humane killing require separate comment. Electrical activity in the brain can persist for up to 30 seconds following these methods [43,44,45,46]; interpretation of the significance of this activity is controversial [47]. As discussed previously, brain electrical activity cannot yet provide definitive answers as to the precise onset of unconsciousness. Other studies [48,49,50,51,52] indicate such activity does not imply the ability to perceive pain and conclude that loss of consciousness develops rapidly.

### 2.4. Plasma and Neuronal Stress Response Markers

#### 2.4.1. Plasma Stress Response Markers 

Both sympathetic nervous system (SNS) and hypothalamic-pituitary-adrenal axis (HPAA) activation are well-accepted as stress response markers. Cortisol and norepinephrine are commonly used to assess levels of stress [53]. In addition, plasma calcium, magnesium, free fatty acids, glucose, and thyroid hormones have also been used to help evaluate stress status [54]. While cortisol levels seem to be unaffected by stunning method in abattoirs [54], the number of negative interactions of pigs with people are positively correlated with plasma cortisol concentrations [55]. Similarly, lactate, a product of glycogenolysis, also increases after stressors such as non-gentle handling [55,56]. Normal pre-slaughter handling increases serum lactate concentrations [57], while plasma lactate concentrations do not return to baseline for 2 hours after stress in pigs [56]. Increased lactate levels may be due to a rise in norepinephrine, as catecholamine release causes an increase in cardiac rate, lowers pH, and produces an accumulation of lactate [58]. Lactate concentrations also increase more quickly after stress than do cortisol concentrations [59]. Hembrecht, *et al.* reported no difference in cortisol concentrations, but reported that catecholamine concentrations were higher in postmortem samples of pigs stunned with CO_2_ than in pigs electrically stunned [58]. However, the procedures were performed at different abattoirs and CO_2_ exerts physiological effects both prior to and following loss of consciousness. There were no differences in plasma concentrations of cortisol, norepinephrine, or lactate post mortem in lambs electrically stunned or stunned with CO_2_ when the concentration of CO_2_ produced effective loss of consciousness [60]. 

Interpretation of plasma stress response markers must consider the effect of animal handling through the entire process, including before, during, and following loss of consciousness. It has been suggested that methods resulting in immediate unconsciousness such as electrical and mechanical stunning, can dramatically increase both epinephrine and norepinephrine levels in samples collected post-stunning [54]. Responses to systemic stressors associated with immediate survival, such as hypoxia and hypercapnia, are likely directly relayed from brainstem nuclei and are not associated with higher order CNS processing and conscious experiences [28]. Marked increases in circulating catecholamines, glucagon, insulin, lactate, and free fatty acids are reported in porcine experimental models where brain death is induced following induction of general anesthesia [61,62,63]. Forslid and Augustinsson reported increased concentrations of norepinephrine and lactate 1 minute after exposure of pigs to 80 percent CO_2_, a level sufficient to produce unconsciousness within 15–30 seconds [59]. Borovsky and coworkers found an increase in norepinephrine in rats following 30 seconds of exposure to 100% CO_2_ [64]. Similarly, Reed and coworkers exposed rats to 20 to 25 seconds of CO_2_, which was sufficient to render them recumbent, unconscious, and unresponsive, and observed 10-fold increases in vasopressin and oxytocin concentrations [65]. Hypothalamic vasopressin-containing neurons are similarly activated in response to CO_2_ exposure in both awake and anesthetized rats [66]. 

Cortisol, lactate, and norepinephrine levels were determined in young pigs undergoing euthanasia with captive bolt or 2 point electrocution (immediate unconsciousness) and inhaled methods (delayed unconsciousness; CO_2_ at displacement rates of 10% and 20% of the chamber volume per min; 70% N_2_/30% CO_2_ at a displacement rate of 20% of the chamber volume per min) [67]. Plasma norepinephrine, and lactate levels were similarly increased with no observed differences between physical or inhaled methods. Although the observed decrease in cortisol with CO_2_ at 10% of the chamber volume per minute displacement rate was statistically different than the increase in cortisol observed with captive bolt or CO_2_ at the displacement rate of 20%, this difference was small (less than 5 µg·dL^−1^) and likely not biologically relevant. Post-weaning plasma cortisol levels in pigs differ by approximately 20 µg·dL^−1^ [68] and the resident intruder test produces cortisol differences of 10 to 15 µg·dL^−1^ [69]. Interestingly, while both CO_2_ and 70% N_2_/30% CO_2_ induced open-mouthed breathing in piglets prior to loss of righting reflex, the mean latency to onset of open-mouth breathing and subsequent loss of righting reflex were substantially longer with 70% N_2_/30% CO_2_ than with CO_2_ [67]. That cortisol, lactate, and norepinephrine levels were similar with both physical and inhaled methods illustrates the difficulty in differentiating conscious from unconscious distress where interpretation is complicated by continued exposure to the inhaled agent during the transitional period between loss of consciousness and death.

#### 2.4.2. C-Fos

Stimulus-induced expression of c-fos mRNA and the Fos protein, products of the c-fos proto-oncogene, are used as markers of stress-induced neuronal activation. Cells respond to external stimuli with a cascade of intracellular events involving the cell membrane, cytoplasm, and nucleus. Within the nucleus, there is a family of proto-oncogenes whose transcription can be activated within minutes; expression of these proto-oncogenes (so-called “immediate-early genes”) precede changes in the expression of other stress-induced genes [70]. Activation is typically brief, with levels rising and disappearing within 2–4 h [71]. 

While neuronal expression of immediate-early genes is a response to many types of stimuli, the *in vivo* expression of the immediate-early genes c-fos and jun-B in the brain is also affected by a variety of general anesthetics. Anesthetics are typically administered with the intent to reduce stress and the physiological effects of restraint and surgery, however they can increase expression of these immediate-early genes and stimulate, rather than suppress, the central nervous system. This enhanced excitability by general anesthetics has been confirmed using an electrophysiological approach [72,73], implying that general anesthetics themselves can induce a type of stress, or a stimulus at least, that evokes some specific intracellular events, rather than suppressing them.

Because of these stimulatory effects, interpretation of stress studies reporting immediate-early gene expression can be difficult in the presence of anesthetics. Up to 90 min may be required for maximal c-fos levels to be observed following brief exposure to a stressor; further, c-fos expression may be localized to highly specific regions of the brain such that local expression using immunostaining may be preferred over more global methods [74]. Similarly, c-fos levels were significantly elevated at 30 min, but not 5 min, after induction and maintenance of anesthesia with isoflurane [75]. Thus, choice and presence of anesthesia, type of stressor, time from exposure to analysis, and assay method may all influence interpretation.

## 3. Conclusions 

Attempting to determine the subjective experience of animals during transitional states of consciousness is very much like the parable of an encounter in the dark with an elephant [76]—while each individual observation may be true, the overall interpretation will be inherently limited. Anesthesia is necessary during these kinds of animal studies for humane and investigational reasons, however anesthetics and the anesthetic state may affect the stress parameters being measured. Drawing from the human experience during transitional states of consciousness is attractive but flawed in that our subjective experience may not coincide with the animal subjective experience. Physiologic observations remain important tools for studying animal stress during transitional states of consciousness provided limitations inherent with these methods are acknowledged and factored into experimental design and interpretation. Use of multiple physiologic stress measures can provide a more complete picture, as can integration with behavior-based observations. In the end, we may only be able to approximate the subjective experiences of animals during transitional states of consciousness.
